# Surgical Treatment of a Giant Proximal Ulnar Artery Aneurysm Potentially Associated With Subacute Endocarditis

**DOI:** 10.7759/cureus.54132

**Published:** 2024-02-13

**Authors:** Vangelis Bontinis, Alkis Bontinis, Argirios Giannopoulos, Vasiliki Manaki, Ioannis Kontes, Kyriakos Ktenidis

**Affiliations:** 1 Vascular Surgery, AHEPA University Hospital, Aristotle University of Thessaloniki, Thessaloniki, GRC

**Keywords:** transesophageal echocardiography, ulnar artery, pseudoaneurysm, subacute, endocarditis

## Abstract

Ulnar artery aneurysms (UAAs), although infrequent, pose limited challenges in terms of timely diagnosis and surgical intervention. Their intricacy lies in discerning and addressing the underlying pathology, often necessitating prolonged hospitalization. Herein, we present a case detailing a giant aneurysm located in the proximal ulnar artery, measuring 5.2 cm in diameter. The patient exhibited negative microbial cultures and non-pathological transthoracic echocardiography (TTE). Successful treatment involved aneurysmal exclusion and saphenous vein graft interposition. While the initial microbiological cultures and TTE yielded negative results, the diagnosis of endocarditis was ultimately confirmed through a subsequent transesophageal echocardiography (TEE) examination. This case report underscores the imperative for heightened clinical suspicion when confronted with upper-limb aneurysms. The diagnostic process necessitates sustained diligence for identifying the underlying pathology, a task that, in certain instances, requires prolonged hospitalization. Both microbiological cultures and TTE have exhibited diminished sensitivity in the diagnosis of infective endocarditis and should consistently be complemented by TEE.

## Introduction

Aneurysmal dilatation of an artery is defined as a 50% increase in the vessel's normal diameter. False aneurysms or pseudoaneurysms manifest as a result of blood extravasation, primarily attributable to vascular injury. Ulnar artery aneurysms (UAAs) are infrequent and predominantly affect the distal segment of the artery. Usually, they are attributed to repetitive microtrauma of the vasculature in the context of occupational activities [1,2]. Infective endocarditis is defined as the infection involving the endothelium of the heart and its valves, with bacteria and fungi identified as the primary pathogens, while it can be acute or subacute [3]. Acute infective endocarditis is characterized by a rapid onset and typical symptoms, while subacute infective endocarditis (SE) unfolds gradually over weeks or months, exhibiting subtle symptoms that can potentially impede its timely diagnosis. Aneurysmal degeneration of the ulnar artery in the setting of SE is extremely rare [4]. We report a case of a giant UAA potentially associated with SE in a patient with a non-pathological transthoracic echocardiography (TTE) and negative microbiological cultures.

## Case presentation

A 63-year-old male presented to our emergency department with a painful, pulsating mass on the inner surface of his right forearm, a condition that had persisted for several months. The patient's medical history revealed no previous traumatic vascular injuries, intravenous drug use, or upper limb procedures involving the puncture of the diseased limb. He did, however, report recent treatment with ampicillin-sulbactam for a respiratory infection.

According to the patient, the initial presentation of the lump was asymptomatic and demonstrated minimal growth, which resulted in a postponed medical consultation. However, two days before his visit, abrupt episodes of pain and a rapid expansion of the mass occurred.

Upon physical examination, brachial, ulnar, and radial arteries were all palpable with a negative Allen's test while the patient was afebrile with stable vital signs. His medical history additionally included the diagnoses of schizophrenia and hypertension, and he self-reported a significant smoking history of 80 pack-years.

**Figure 1:**
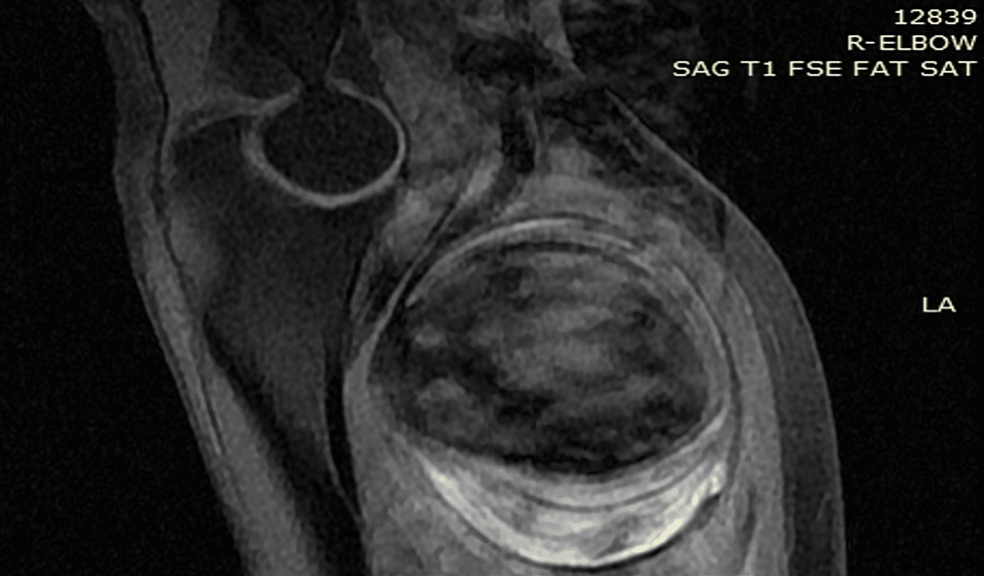
MRI of the right forearm

Laboratory findings included leukocytosis (WBC: 19.67 k/μl, neutrophils: 90.6%) and normocytic anemia (hemoglobin: 9.1 g/dL, hematocrit: 28.3). Furthermore, concerning the findings of anemia, the patient denied experiencing hematemesis, hematochezia, melena, or any presence of blood in the stool. His electrocardiography (ECG) findings revealed a sinus rhythm with right bundle branch block (RBBB) while his chest x-ray showed no evidence of pathological lesions. Subsequent examinations, including color Doppler ultrasound and upper-arm MRI, concurred in depicting a brachial artery aneurysm measuring 6.6 x 5.2 cm in diameter and 7.2 cm in length at the level of the brachial artery bifurcation (that was the initial diagnosis made by the radiology department) (Figure 1).

**Figure 2:**
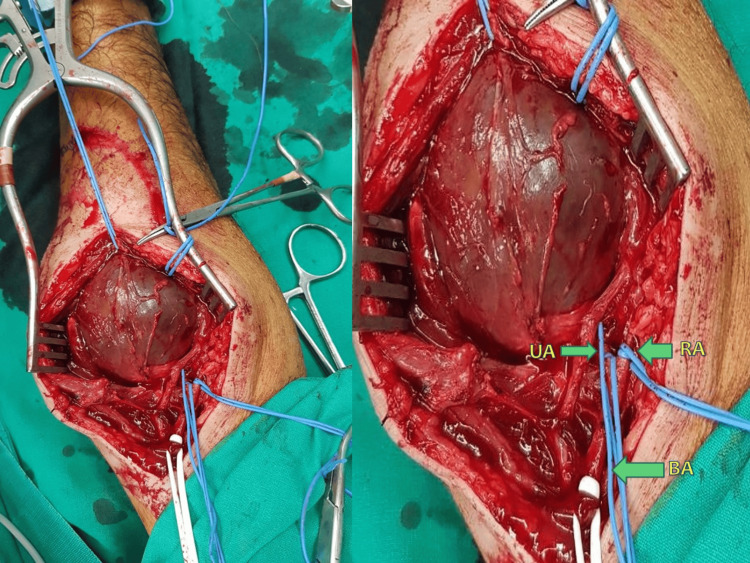
Surgical exposure of UAA UA: Ulnar artery, RA: Radial artery, BA: Brachial artery; UAA: Ulnar artery aneurysm

The patient was transferred to the operating room, where aneurysm resection under local anesthesia was conducted. A longitudinal incision was made on the inner surface of the right arm, extending from the antecubital fossa down to the forearm, exposing a sizable aneurysm. Surgical exploration at the brachial artery bifurcation level revealed the true origin of the aneurysm, situated in the proximal segment of the ulnar artery. The aneurysm closely adhered to the median nerve and the radial artery, necessitating meticulous surgical dissection (Figure 2). Tissue specimens were obtained for subsequent microbiological examination. Aneurysmal exclusion was performed, followed by saphenous vein graft interposition and an end-to-end anastomosis.

On the first postoperative day, laboratory findings showcased a dramatic decrease in leukocyte count (WBC: 10.2 k/μl). Moreover, TTE was performed with negative findings for heart lesions, including valvular vegetations.

Given that our institution was functioning as a COVID-19 referral hospital at the time, efforts were made to minimize the duration of hospitalization. The next day, the patient was referred to the regional hospital in his district for further evaluation of his anemia and leukocytosis. Unfortunately, the patient chose not to follow our guidance and returned home, continuing the prescribed treatment regimen, which included oral antibiotics (ampicillin-sulbactam), acetylsalicylic acid (100 mg daily), and tinzaparin (4,500 units daily) for a subsequent two-week period until re-evaluation. Concurrently, the results of the microbiological culture of the tissue specimen returned negative.

One week post discharge, we received notification about the patient's urgent transfer to his district's local hospital due to elevated fever. Upon thorough assessment, the patient received a diagnosis of infective endocarditis, supported by the identification of heart valve vegetations during transesophageal ultrasound. Notably, the patient's right arm demonstrated optimal condition, maintaining full functionality, and exhibited no indications of neuralgia or paresis.

## Discussion

UAAs are rare yet significant arterial lesions necessitating prompt diagnosis. These lesions may lead to digital ischemia or necrosis and, in more severe cases, result in arterial rupture with potentially catastrophic consequences. A multitude of etiologies has been documented, encompassing vascular anomalies, vasculitis, infections, eosinophilia, and endocarditis.

As outlined by Kuntz et al., the scarcity of reports on upper limb aneurysms precludes the establishment of definitive conclusions concerning their optimal treatment, particularly in cases where aneurysms are situated more proximally to the brachial artery. Moreover, post-revascularization complication rates of approximately 10% evoke skepticism [5]. In our case, we elected to undertake revascularization employing an autologous vein graft, given the patient's relatively young age, and considering that the affected limb constituted the dominant arm.

Aneurysmal degeneration secondary to endocarditis occurs subsequent to septic embolization, wherein the inflammatory process permeates the arterial wall, resulting in the formation of a mycotic aneurysm [6]. The diagnosis is often challenging, requiring high clinical suspicion, whereas it is based on the combination of clinical symptoms, microbiological specimens and imaging modalities.

In the context of our diagnostic protocol, the patient's microbial culture produced negative results, and TTE disclosed the absence of heart valve vegetations. Furthermore, in the absence of obtaining blood cultures due to the patient's afebrile condition upon presentation, it became evident that the patient satisfied only one minor criterion that of 'vascular phenomena,' among Duke's criteria for diagnosing infective endocarditis. These criteria encompass predisposing cardiac lesions, fever exceeding 38°C, intravenous drug use, embolic phenomena, and immunologic phenomena [7].

In retrospect and despite low clinical suspicion, it may have been advisable to augment TTE with transesophageal echocardiography (TEE). This consideration arises from the recognized high incidence of false-negative results associated with TTE. Notably, TTE's sensitivity for detecting endocarditis is approximately 75%, with reported rates as low as 24% [8,9].

Furthermore, the absence of positive microbial cultures should not instill reassurance, given that approximately 23% of blood/tissue cultures in the context of infective endocarditis return negative results. This holds particular significance when patients are undergoing antibiotic therapy at the time of the collection of the microbiological specimen, as was exemplified in the case of our patient.

A handful of reports regarding infective endocarditis and UAA can be found in the literature. To our knowledge, there is only one publication regarding UAA due to SE, while this is the first publication regarding proximal UAA potentially associated with SE. In their publication, Shamsolkottabi et al. present a case of a mycotic aneurysm of the distal ulnar artery (hypothenar region) successfully treated with surgical excision in a patient with known SE [4].

While rational arguments opposing our hypothesis linking the UAA to SE may exist, two crucial points merit consideration. Firstly, the development of heart valve vegetations can span several months, whereas our patient received a diagnosis merely one week post aneurysmal treatment, rendering the emergence of vegetations within such a brief time frame highly improbable. Secondly, it is pertinent to acknowledge that infective endocarditis is attributed to a spectrum of pathogens. However, in this particular case, we can only speculate that the initiation of SE potentially coincided with the patient's pneumonia diagnosis [10].

The definitive classification of the aneurysm observed in this study as a mycotic aneurysm remains uncertain, primarily due to negative cultures and the absence of histologic examinations on the obtained specimens. Nonetheless, considering the close correlation between the two diagnoses and the provided information, it is justifiable to entertain the suspicion of a mycotic aneurysm associated with subacute endocarditis.

## Conclusions

This case report underscores the imperative of maintaining a heightened clinical suspicion when confronted with upper-limb aneurysms. Although surgical correction frequently results in satisfactory outcomes, the diagnostic process necessitates persistence in uncovering the latent underlying pathology. In certain scenarios, this necessitates prolonged hospitalization to enable a comprehensive investigation.
